# Epidemics

**Published:** 1854-10

**Authors:** 


*Epidemics.*—The cholera seems, at this writing, to have disappeared entirely from Buffalo. Very few deaths occurred during the third week of of September, and at the beginning of the fourth we hear of no new cases.

The same is true of most of the cities in which it has prevailed. Very few of the principal towns of the country have entirely escaped; and some, as in the instances of Rochester and Pittsburg, after passing a healthy summer, have suffered very severely at the close of the season.

In Buffalo, at a late period of the epidemic, a locality on South Division street, apparently healthy, and inhabited by a very good class of citizens, became the seat of disease to a considerable extent. It was confined almost entirely to a single square, and was very malignant in its character. What may have been the local cause is unknown, though there can be little doubt that such a cause existed.

The influence of local causes has been strikingly exhibited during the season in this city. We have already said so much upon this subject, that we need press it no farther than to say, that those who have committed themselves to any single agent as an exciting cause of cholera, must have found it very difficult, this year, to harmonize their theory with their facts. It is more than ever evident that any exhausting agency is a cholera cause.

The letter from "Clericus, Jr.," which occupies another page of the editorial department, may seem to call for some rejoinder from us.

"Clericus" should bear in mind, that he is not original in his treatment of cholera by calomel alone. It is a remedy which in former years has attracted much attention, and been the subject of discussion. So far as we know, the opinion of those who have had best opportunity for judging, is not especially in its favor. We should not expect to obtain from it, *in a series of cases*, the success of our correspondent. At the same time the four cases of "Clericus, Jr.," should be admitted as instructive facts, and good arguments, so far as they go, for the exclusive calomel treatment, inasmuch as they come from a source entirely reliable.

Many others would be able to show as good a success from the pure opium treatment, were they to take a limited number of their cases. Whatever may have been the differences of treatment, it is evident that we are not behind other countries in success. The average of deaths in Paris and many other European cities has been much greater than in most American towns.

